# Genome Dominance in *Allium* Hybrids (*A. cepa* × *A. roylei*)

**DOI:** 10.3389/fpls.2022.854127

**Published:** 2022-03-10

**Authors:** David Kopecký, Olga Scholten, Joanna Majka, Karin Burger-Meijer, Martin Duchoslav, Jan Bartoš

**Affiliations:** ^1^Institute of Experimental Botany, Czech Academy of Sciences, Center of the Region Hana for Biotechnological and Agricultural Research, Olomouc, Czechia; ^2^Plant Breeding, Wageningen University and Research, Wageningen, Netherlands; ^3^Institute of Plant Genetics, Polish Academy of Sciences, Poznań, Poland; ^4^Department of Botany, Palacký University, Olomouc, Czechia

**Keywords:** onion, meiotic drive, interspecific hybridization, homoploid, female meiosis, genome stability, homoeologous recombination

## Abstract

Genome dominance is a phenomenon in wide hybrids when one of the parental genomes becomes “dominant,” while the other genome turns to be “submissive.” This dominance may express itself in several ways including homoeologous gene expression bias and modified epigenetic regulation. Moreover, some wide hybrids display unequal retention of parental chromosomes in successive generations. This may hamper employment of wide hybridization in practical breeding due to the potential elimination of introgressed segments from progeny. In onion breeding, *Allium roylei* (*A. roylei*) Stearn has been frequently used as a source of resistance to downy mildew for cultivars of bulb onion, *Allium cepa* (*A. cepa*) L. This study demonstrates that in *A. cepa* × *A. roylei* hybrids, chromosomes of *A. cepa* are frequently substituted by those of *A. roylei* and in just one generation, the genomic constitution shifts from 8 *A. cepa* + 8 *A. roylei* chromosomes in the F1 generation to the average of 6.7 *A. cepa* + 9.3 *A. roylei* chromosomes in the F2 generation. Screening of the backcross generation *A. cepa* × (*A. cepa* × *A. roylei*) revealed that this shift does not appear during male meiosis, which is perfectly regular and results with balanced segregation of parental chromosomes, which are equally transmitted to the next generation. This indicates that female meiotic drive is the key factor underlying *A. roylei* genome dominance. Single nucleotide polymorphism (SNP) genotyping further suggested that the drive has different strength across the genome, with some chromosome segments displaying Mendelian segregation, while others exhibiting statistically significant deviation from it.

## Introduction

Introgression breeding is the way to efficiently transfer agronomically beneficial alleles from wild relatives to crops. This involves interspecific mating followed by one or more rounds of backcrossing to the recipient parent. Many traits have been improved *via* introgression breeding, including resistance to pests and diseases, tolerance to abiotic stresses, and root-related traits (Anamthawat-Jónsson, 2001; Scholten et al., 2007; Placido et al., 2013; Molnár-Láng, 2015). However, introgression lines frequently suffer from the instability of the introgressed segment(s) in the successive generations (Kopecký et al., 2019; Pernickova et al., 2019). Combining two genomes in a single nucleus, it opens a way for genome dominance, a phenomenon, when one parental genome becomes dominant, while the other tends to be submissive in a hybrid progeny. Such dominance can manifest itself in several ways, including altered gene expression and epigenetic regulation (Glombik et al., 2020). Most, if not all, allopolyploids retain the expression level of one (dominant) parent (so-called expression level dominance) and/or display a preferential expression from the alleles of the dominant genome (so-called homoeolog expression bias). Such dominance does not involve all the expressed genes, as some genes can be overexpressed from the submissive genome or display the overall expression at the level of the submissive parent (Edger et al., 2017; Bird et al., 2018; Glombik et al., 2021).

Another expression of genome dominance is elimination of chromosomes of the submissive genome or their replacement by those of the dominant genome (Glombik et al., 2020; Majka et al., 2020). Chromosome elimination usually occurs in hybrids where there is no pairing of homoeologous chromosomes (i.e., chromosomes from two more or less distinct parental species). Restriction of homoeologous pairing can be a consequence of either DNA sequence dissimilarity, which precludes homoeologous recognition and initiation of pairing during prophase I of meiosis or the action of a molecular mechanism preventing dissimilar DNA sequences from forming crossovers. The textbook example of such a mechanism is *pairing homoeologous 1* (*Ph1*) in wheat (Sears and Okamoto, 1958), which is also capable of modifying chromosome pairing of other species when transferred from wheat (Lukaszewski and Kopecky, 2010). In wheat-rye hybrids, rye chromosomes are more prone to elimination during meiosis, despite strict homoeologous chromosome pairing (Tsunewaki, 1964; Lukaszewski et al., 1987; Pernickova et al., 2019).

While considered rare in the past, wide hybrids with (extensive) homoeologous chromosome pairing readily develop in nature and can also be created artificially, for example, as part of breeding programs. Chromosomes of ryegrass (*Lolium* spp.) pair and recombine freely with those of fescue (*Festuca* spp.) in xFestulolium hybrids (Kopeckyì et al., 2008; Zwierzykowski et al., 2008). Similarly, various hybrids of ornamental plants, such as lily hybrids, *Alstroemeria aurea* × *Alstroemeria inodora* and *Gasteria lutzii* × *Aloe aristata*, show homoeologous chromosome pairing (Takahashi et al., 1997; Kamstra et al., 1999; Karlov et al., 1999; Khan et al., 2009). The ability of homoeologous chromosomes to pair in meiosis opens the way for competition between parental chromosomes. While male meiosis is symmetrical with all the four products producing gametes that can contribute to the successive generation, female meiosis is asymmetrical where only one product generates a gamete (the egg cell), while the genetic information in the remaining cells is not passed onto the next generation. This aspect of female meiosis creates an opportunity for chromosome competition and this phenomenon is called “meiotic drive” (Sandler and Novitski, 1957). In hybrid (homoploid) mice with regular (homoeologous) chromosome pairing, Akera et al. (2017) observed biased orientation of parental chromosomes on the karyokinetic spindle during female meiosis. Chromosomes from the dominant genome tended to orient toward the pole eventually producing the egg cell more frequently than those from the submissive genome and their frequency among progeny was higher than expected from random segregation.

As mentioned above, interspecific hybridization is often used in breeding programs to introgress one or more desired traits in crops, usually from wild relatives. Using this approach, lines of cultivated bulb onion *Allium cepa* (*A. cepa*) L. with chromosome segments carrying downy mildew [*Peronospora destructor* (Berk.) Casp.] resistance gene(s) introgressed from its wild relative *Allium roylei* (*A. roylei*) Stearn. were developed by research programs of Wageningen University and Research Center (Netherlands) and Russian State Agrarian University (Russia) (van der Meer and de Vries, 1990; Khrustaleva and Kik, 1998; Khrustaleva et al., 2019). A combination of phenotyping, genotyping with DNA markers, and cytogenetic analyses of advanced backcross (BC1) generations allocated the putative downy mildew resistance locus *Pd1* to the region spanning the most distal ∼18% of the long arm of chromosome 3 (van Heusden et al., 2000). By controlled intercrosses and BC1, homozygous introgression lines were obtained that were resistant to downy mildew. Reduction of the introgressed segment length was an important step further, also because of a recessive lethal factor located in a close vicinity of the *Pd1* locus and probably expressed only in the *A. cepa* background (Scholten et al., 2007; Kim et al., 2016; Khrustaleva et al., 2019). Separating that lethal factor from *Pd1* gene by crossing over, it was observed in a single plant out of 215 plants screened, suggesting a tight linkage (Scholten et al., 2007). *A. roylei* has also been proposed to use as a bridge for introgression of traits from the Welsh onion [*Allium fistulosum* (*A. fistulosum*) L.] into cultivated bulb onion, as direct introgression is difficult because of a very low fertility of the hybrids between bulb onion and Welsh onion (Khrustaleva and Kik, 1998, 2000; Stevenson et al., 1998; Budylin et al., 2016).

The main aim of this study was to evaluate the stability of the newly established hybrid genome in hybrids of *A. cepa* × *A. roylei*, to shed light on the mechanisms underlying possible genome dominance, and to estimate the retention rate of individual chromosomal segments of *A. roylei* and *A. cepa* in successive hybrid generation(s).

## Materials and Methods

### Plant Material

A total of 104 F2 plants from a cross *A. cepa* ♀ × *A. roylei* ♂ were analyzed in this study. The F2 population was generated by selfing of one plant of the interspecific F1 (CxR) (PRI 93103), a hybrid genotype between *A. cepa* and *A. roylei*. Of those 104 plants, 75 plants were analyzed by genomic *in situ* hybridization (GISH) (to determine their genomic constitution). All the plants were genotyped by single nucleotide polymorphism (SNP) markers, of which 80 plants were included earlier in the production of a linkage map (Scholten et al., 2016). Part of the F2 family was the original population used in the study of van Heusden et al. (2000) and all the plants shared the same parental lineages. In addition, GISH was used to analyze the genome composition of 21 BC1 plants produced by a BC1 of the F1 hybrid used as a pollinator with a male-sterile *A. cepa*.

### Chromosome Preparations and Genomic *in situ* Hybridization

Roots of individual plants were collected and their cell cycle was synchronized using iced distilled water for 28 h following fixation in Carnoy’s solution (absolute ethanol/glacial acetic acid, 3:1 v/v). For meiotic analyses, flower buds were fixed in Carnoy’s solution at 37°C for 7 days. Individual anthers that were confirmed to be in the proper meiotic stage were squashed in a drop of acetic acid and used for GISH. Chromosome preparations were made according to a study by Masoudi-Nejad et al. (2002). GISH analyses were done on the mitotic and meiotic chromosome spreads according to a study by Ferreira et al. (2021). Total genomic DNA (gDNA) of *A. cepa* was used as blocking DNA and total gDNA of *A. roylei* was labeled with digoxigenin (DIG) using the DIG-Nick Translation Kit (Roche Applied Science, United States) according to the manufacturer’s instructions and used as a probe. The probe/blocking DNA ratio was ∼1:150. Signal detection was made with anti-DIG-fluorescein isothiocyanate (FITC) conjugate (Roche Applied Science). Chromosomes were counterstained with 4′,6-diamidino-2-phenylindole (DAPI) in Vectashield (Vector Laboratories, Oberkochen, Germany). Chromosome analyses were done using an Olympus AX70 microscope equipped with epifluorescence and a SensiCam B/W camera. Images were captured with Microimage software and processed with Adobe Photoshop version 6 software (Adobe Systems Corporation, San Jose, CA, United States). The proportions of *A. roylei* and *A. cepa* chromosomes in hybrids were tested against the assumption of a 1:1 ratio representing Mendelian inheritance. The number of *A. roylei* chromosomes was expressed as the proportion *p* of the total number of chromosomes within the cell and H_0_:*p* = 0.50 was tested by the one-sample *t*-test in R (R Core Team, 2021).

To determine the positions of the crossovers along chromosomes in F2 and BC1 hybrids, we measured the lengths of introgressed segments and the lengths of both the arms of recombined chromosomes using the Scion Image software (Scion Corporation, Frederick, MD, United States). The difference in the distribution of crossovers between male meiosis and both the meioses was evaluated by comparing their distributions along chromosome arms divided into 10 segments (bins) of 10% of their length. Two empirical distributions were compared using the function ks.boot in the Matching library in R. The function uses a bootstrap version of the Kolmogorov–Smirnov test, providing accurate coverage even when the distributions being compared are not entirely continuous and ties occur in the dataset (Sekhon, 2011).

A total of 50 pollen mother cells (PMCs) were evaluated in each of the meiotic stages (prophase I, metaphase I, anaphase I, and telophase I) in a single F1 *A. cepa* × *A. roylei* plant (PRI 93103).

### Single Nucleotide Polymorphism Genotyping

The 104 F2 hybrids were genotyped using SNPs markers (all the 75 plants used for GISH karyotyping and 29 others from the same cross), as described previously (Scholten et al., 2016). Only markers enabling unambiguous discrimination of *rr* (homozygote for *A. roylei* allele), *rc* (heterozygote), and *cc* (homozygote for *A. cepa* allele) genotypes were selected. The statistically significant deviation of frequencies of three genotype classes in F2 hybrids from the theoretical 1 *rr*:2 *rc*:1 *cc* of Mendelian inheritance (H_0_) was assessed using multinomial test in R, separately for each SNP marker.

## Results

### Chromosomes of *A. cepa* Are Replaced by Those of *A. roylei* in F2 Hybrids

**FIGURE 1:**
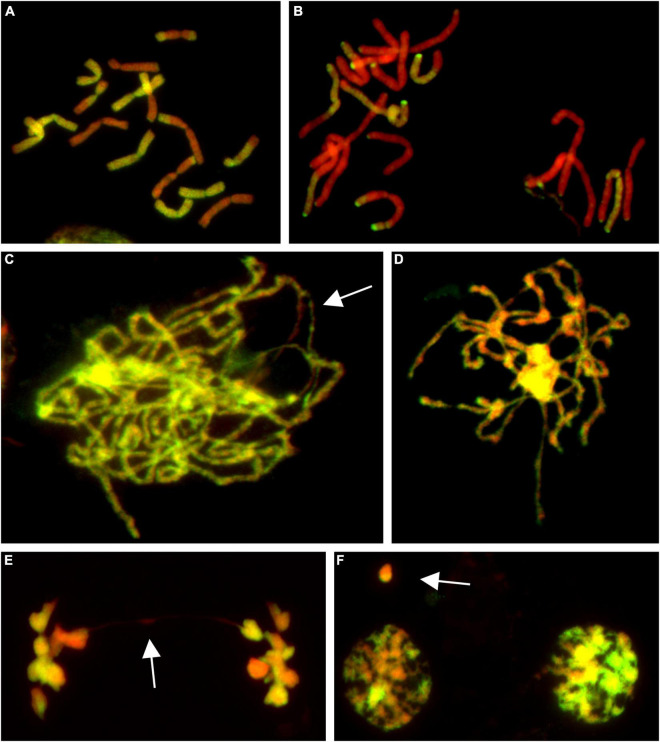
Molecular cytogenetic analysis of *Allium cepa* (*A. cepa*) × *Allium roylei* (*A. roylei*) hybrids. Mitotic cells of F2 **(A)** and backcross (BC1) **(B)** plants and meiotic cells of F1 hybrid **(C–F)** after genomic *in situ* hybridization (GISH). During meiosis, homoeologous chromosomes initiate pairing in zygotene [**(C)**; so far unpaired segments indicated by arrow] with complete pairing in pachytene **(D)**. During anaphase I, chromosomes segregate to opposite poles **(E)** with only rare bridges (arrow) forming diads in TI **(F)** with rare micronuclei (arrow). Total guide DNA (gDNA) of *A. roylei* was labeled with digoxigenin (green/yellow color) and sheared DNA of *A. cepa* was used as blocking DNA (red pseudocolor).

**FIGURE 2:**
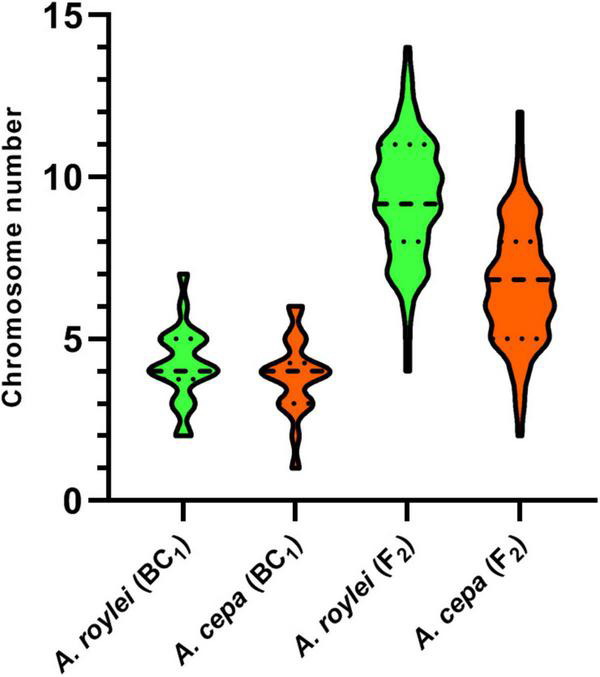
Genome composition of F2 hybrids of *A. cepa* × *A. roylei* and genome composition of the male gametes (pollen grains) calculated from the genome composition of BC1 progeny of *A. cepa* × (*A. cepa* × *A. roylei*). Number of parental chromosomes is based on the origin of the centromere region of a particular chromosome. A dashed line in a violin plot represents median and dotted lines represent quartiles.

Among 75 individuals of the F2 generation, we detected a significant shift in genome composition from eight chromosomes of *A. cepa* plus eight chromosomes of *A. roylei* in the F1 genotype toward the *A. roylei* genome. However, homoeologous crossover events occurred complicating classification of parental chromosomes/genomes. Thus, we consider the origin of the chromosome based on the fluorescence signal spanning its centromeric region (Figure 1A). On average, there were 9.33 chromosomes of *A. roylei*—4.07 complete and 5.26 recombined (homoeologous recombination) and 6.67 chromosomes of *A. cepa*—1.69 complete and 4.97 recombined (Figure 2). This proportion of *A. roylei* and *A. cepa* chromosomes significantly deviated from the 1:1 ratio (two-sided one sample *t*-test, mean proportion *p* of *A. roylei* chromosomes ± SD: 0.58 ± 0.12, *t* = 6.11, *df* = 74, *P* < 0.001) and roughly accounts for the ratio of 1.4:1 of *A. roylei* vs. *A. cepa* centromeres (Supplementary Table 1).

### Male vs. Female Meiosis

We analyzed the consequence of male meiosis by screening the BC1 generation (male sterile *A. cepa* ♀ × F_1_ hybrid ♂). Female meiosis could not be assessed in the same fashion, as all the attempts to produce seed from the reciprocal BC1 (F_1_ hybrid ♀ × *A. cepa* ♂) failed. Therefore, the contribution of female meiosis can only be assessed by subtraction of the detected effects of male meiosis from the combined contribution of both the sexes to the F2 generation. The number of crossovers per bivalent calculated from the frequency of recombined chromosomes among progeny was about the same: 1.81 ± 0.48 (mean ± SD) in male meiosis and 1.65 ± 0.38 in both the meioses (two-sided equal-variance *t*-test, *t* = −1.51, *df* = 91, *P* = 0.133). Similarly, the difference in numbers of crossovers per recombined chromosome was also non-significant: 1.35 ± 0.24 in male meiosis vs. 1.29 ± 0.16 in both the meioses (two-sided Aspin–Welch unequal-variance *t*-test, *t* = −1.08, *df* = 20.74, *P* = 0.292). It, therefore, appears that the recombination rate in male and female meiosis was the same (not different).

**FIGURE 3:**
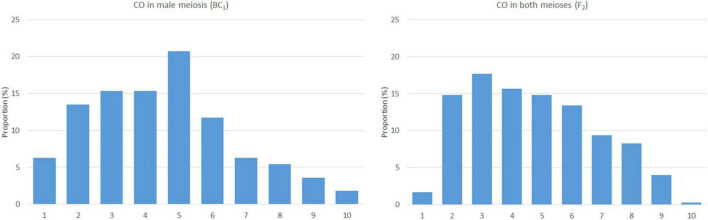
The frequency and distribution of crossovers in male (left) and both the meioses (right). The x-axis represents a chromosome arm (from the telomere on the left to the centromere on the right) divided into bins of 10% of relative arm length.

Based on the lengths of chromosome segments in recombined chromosomes and the positions of the crossover points, we were able to estimate the distribution of recombination events along the chromosomes. We did not include double crossovers (two crossovers in a single arm), as these are subjected to crossover interference (Ferreira et al., 2021) and may bias the overall results. However, two crossovers per chromosome, one in each arm, were included, as the centromere does act as a barrier to crossover interference. In this material, arms of a chromosome appear to be independent units in the process of crossing over (Ferreira et al., 2021). The plants of the F2 generation (contribution of both the meioses) show a pattern of homoeologous recombination distributed unevenly along chromosomes, with a higher frequency in distal regions, except for the terminal bin and highly reduced frequency in proximal regions around centromeres. A reduction in homoeologous recombination in (sub)telomeric and (peri)centromeric regions was also observed in BC1 plants (male meiosis); the highest frequency was found in interstitial regions of the chromosome arms (Figure 3). The difference between male and both the meioses in the distribution of homoeologous recombination was statistically significant (two-sided two-sample Kolmogorov–Smirnov test, *D* = 0.7, bootstrap *P* = 0.007).

### Male Meiosis Does Not Introduce the Bias in Genome Composition

Male meiosis of F1 seems to be regular with the formation of bivalents consisting of homoeologous chromosomes during prophase I (Figures 1C,D) and metaphase I. Ring bivalents predominated over rod bivalents (5.06 vs. 2.94 per cell), suggesting 1.63 crossovers per bivalent. In anaphase I, segregation of homoeologous chromosomes toward opposite poles without lagging chromosomes was observed and we observed a chromosome bridge in only one out of 50 cells (Figure 1E). Similarly, micronuclei were observed in only two cells out of 51 cells in telophase I, one being of *A. cepa* origin and the other seemed to be composed by chromatin of both the progenitors, probably as a result of homoeologous recombination (Figure 1F). These results demonstrate that male meiosis is fairly regular (Supplementary Figure 1).

The analysis of the BC1 (*A. cepa* ♀ × F_1_ hybrid ♂) provided us with an estimate of the effect of male meiosis on the genome composition of the progeny in *A. cepa* × *A. roylei* hybrids (Figure 1B). As the gamete (egg) of *A. cepa* had eight chromosomes of *A. cepa*, remaining chromosomes of the progeny had to come from male gamete (pollen grain) of the F1 hybrid. Once the F1 hybrids have eight chromosomes of *A. cepa* and eight chromosomes of *A. roylei*, a theoretical average constitution of a pollen grain is four chromosomes of *A. cepa* + four chromosomes of *A. roylei*. This is close to what we found among 21 plants of the BC1 generation: 11.86 chromosomes of *A. cepa* (eight of them being from the egg of *A. cepa*) and 4.19 chromosomes of *A. roylei* (Figure 2). With the assumption that female gamete contributed to the embryo with eight *A. cepa* chromosomes, the average genome composition of pollen grain is to be 3.86 *A. cepa* and 4.19 *A. roylei* chromosomes. The difference between the observed ratio and theoretical 1:1 ratios of Mendelian inheritance was non-significant (two-sided one-sample *t*-test, mean proportion *p* of *A. roylei* chromosomes ± SD: 0.52 ± 0.14, *t* = 0.63, *df* = 20, *P* = 0.537). While 20 plants were euploid with 16 chromosomes, one plant was aneuploid with 17 chromosomes. These results indicate that male meiosis produces viable gametes with almost equal proportions of parental chromosomes and does not significantly contribute to *A. roylei* genome dominance. By subtraction, it appears that female meiosis is likely the driving force of this phenomenon. Considering 3.86 chromosomes of *A. cepa* and 4.19 chromosomes of *A. roylei* transmitted by the pollen grain, the average egg cell must have contributed 2.81 *A. cepa* and 5.14 *A. roylei* chromosomes to achieve genome composition observed in the F2 hybrids (6.67 chromosomes of *A. cepa* + 9.33 chromosomes of *A. roylei*).

### Genome Dominance Seems to Be Chromosome Specific

Single nucleotide polymorphism genotyping of 104 progeny provided another measure of the genome composition of the F2 hybrids along all the eight linkage groups representing individual chromosomes [based on genetic map of this population previously published as supplemental data by Scholten et al. (2016)]. We selected 119 SNP markers, which clearly distinguished all the three genotype classes: *rr* (homozygote for *A. roylei* allele), *rc* (heterozygote), and *cc* (homozygote for *A. cepa* allele) distributed over all the eight chromosomes (ranging from 8 to 24 per chromosome).

Unfortunately, positions of centromeres are not known on the genetic maps, which hamper direct comparison of GISH analysis and SNP genotyping. However, it is evident that results from SNP genotyping are in line with those from GISH: we observed 3,647 *rr*, 6,749 *rc*, and 1,691 *cc* genotypes. This can be translated into the ratio (3,647 × 2) + 6,749 *r* allele vs. (1,691 × 2) + 6,749 *c* allele or a ratio of 1.39 *r*:1 *c*. This is almost identical to the ratio obtained from the GISH results of centromeres (1.4:1), suggesting high fidelity of our results. However, the variability in the genome composition revealed by SNP genotyping was large between individual plants and ranged from 78.2 *r*:21.8 *c* (roughly 3.6:1) to 32.8 *r*:67.2 *c* (roughly 1:2).

**FIGURE 4:**
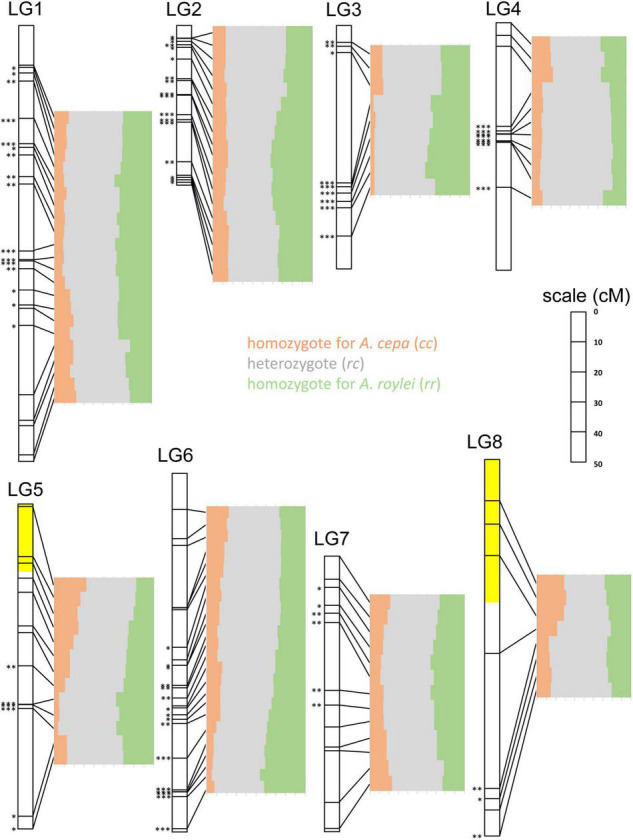
Segregation of *A. cepa* and *A. roylei* alleles in F2 generation. Application of single nucleotide polymorphism (SNP) markers enabled visualization of segregation distortion of the regions along individual chromosomes [linkage groups based on genetic map of Scholten et al. (2016)]. Statistically significant deviation from the Mendelian 1:2:1 ratio of *rr*:*rc*:*cc* genotypes was tested by multinomial test, separately for each SNP marker (^∗^*P* < 0.05; ^∗∗^*P* < 0.01; ^∗∗∗^*P* < 0.001). Two regions showing (statistically non-significant) distortion toward *A. cepa* alleles are highlighted with yellow color.

When focusing on individual chromosome regions along the entire genomes, we found high variability in the genome composition and frequent statistically significant deviations from the Mendelian 1:2:1 ratio of *rr*:*rc*:*cc* genotypes as tested by multinomial test, separately for each SNP marker (Figure 4).

LG1: Entire chromosome displayed genome dominance of *A. roylei*. Distal parts of the chromosome showed non-significant deviation, while the segment between 18 and 81 cM was deviated significantly from the theoretical 1:2:1 in favor of the *rr* and *rc* genotypes (*P* < 0.01).

LG2: All but one marker showed a statistically significant deviation (*P* < 0.01) from the theoretical ratio of 1:2:1 toward the *rr* and *rc* genotypes.

LG3: There was a strong deviation from the 1:2:1 toward the *rr* and *rc* genotypes (*P* < 0.01 for eight out of nine markers).

LG4: There was a non-significant difference from 1:2:1 in the distal part of one arm (from 0 to 8 cM), while all the remaining segments of the chromosome displayed a shift toward *rc* heterozygote constitution (*P* < 0.001).

LG5: Approximately, one-half of the linkage group showed a non-significant deviation, while the other half displayed a significant deviation (*P* < 0.01) in favor of *rr* and *rc* genotypes. Interestingly, the distal part of the chromosome represented by three markers (from 0 to 20 cM) was one out of two regions of the genome displaying higher number of *cc* genotypes than *rr* genotypes (but the deviation is non-significant).

LG6: Approximately, one-half of the linkage group (from 0 to 64 cM) showed a non-significant deviation from the theoretical, while other half significantly deviated (*P* < 0.05) toward the *rr* and *rc* genotypes. A strong deviation (*P* < 0.001) was found at the distal part of the chromosome (from 95 to 120 cM).

LG7: Distal regions showed no significant deviation, while the interstitial part (from 10 to 50 cM) deviated from 1:2:1 toward the *rr* and *rc* genotypes (*P* < 0.05).

LG8: Three out of eight markers in the distal region of one arm show an excess of the *cc* genotypes (non-significant deviation from 1:2:1), while other parts deviated significantly (three markers) or non-significantly (two markers) from theoretical 1:2:1 toward the *rr* and/or *rc* genotypes.

## Discussion

Cultivated crops are usually limited in diversity, by the domestication bottleneck and long-lasting selection. Thus, introgression of genetic diversity from alien sources, in general, or of alleles of agronomically beneficial loci never present in a crop or lost during the evolution and/or selection is a step toward the development of superior cultivars. In bulb onion (*Allium cepa*) breeding, alleles for downy mildew [caused by *Peronospora destructor* (Berk.) Casp.], leaf blight (caused by *Botrytis squamosa* Walker), and anthracnose [caused by *Colletotrichum gloeosporioides* (Penz.) Penz. and Sacc.] resistance can be introgressed from a wild relative, *A. roylei* (Kofoet et al., 1990; de Vries et al., 1992; Galvan et al., 1997; Scholten et al., 2016). However, alien introgressions are not always stable in the host genome of a crop and may be lost over generations. Various studies indicate that merging two genomes from different species results in massive changes at different levels, including modifications of gene expression and epigenetic regulation, genome down- or upsizing, and chromosome reshuffling (Wendel, 2015; Van de Peer et al., 2021). In many hybrids, one of the parental genome becomes “dominant,” whereas the other turns to be “submissive.” Such genome dominance can be expressed at various levels including elimination of chromosomes from the submissive genome or replacement of such chromosomes by those from the dominant genome (Glombik et al., 2020). In triticale, a hybrid of wheat and rye, chromosomes of rye are more prone to elimination than their wheat counterparts, which may lead to the reversion to pure wheat forms in successive generations (Orellana et al., 1984). In xFestulolium, a hybrid of ryegrass (*Lolium* spp.) and fescue (*Festuca* spp.), chromosomes of *Festuca* are gradually replaced by those of *Lolium* (Kopeckyì et al., 2006; Zwierzykowski et al., 2006). In introgression cultivars, complete elimination of *Festuca* segments is expected to happen within 3–4 generations of multiplication (Kopecký et al., 2019). Therefore, studies on the genome stability of the hybrid genomes and the transmission of the introgressed segment(s) to successive generations may offer some guidance in assessing the potential of the introgression breeding.

In hybrid onion *A. cepa* × *A. roylei*, homoeologous chromosomes pair and recombine, but there is only scarce information on the genome dominance. When compiling genetic maps of the parental species *via* genotyping of F2 hybrids (from the same cross as those in this study) with amplified fragment length polymorphism (AFLP) markers, van Heusden et al. (2000) mentioned that the *cepa*-specific markers were not amplified in 28% of the F2 plants compared to 16% of the *roylei*-specific markers. From that, the authors estimated that the contribution of the *cepa*-specific and *roylei*-specific alleles in the F2 generation was about 44 and 56%, respectively. This is in line with the results obtained in this study, where results from SNP genotyping indicate the proportion of 42–58%, respectively, while GISH karyotyping revealed 42% of centromeres being of *A. cepa* origin and 58% of centromeres being of *A. roylei* origin. These results indicate violation of the Mendelian law of random segregation. However, the *roylei* genome dominance was not observed in all the plants and much variation was observed between individual plants, with the percentage of *cepa*-specific alleles ranging from 15 to 68% (van Heusden et al., 2000). Similar variation was found in this study: the frequency of *c* alleles ranged from 22 to 67% among individual plants. The correspondence of our results from GISH and SNP genotyping with AFLP markers of a study by van Heusden et al. (2000) indicates that the genome dominance is consistent at the level of about 42:58 in *A. cepa* × *A. roylei* hybrids. However, studies on different wide hybrids provided different results, ranging from synthetic *Brassica napus* (*B. rapa* × *B. oleracea*) with proportion of parental genomes 43:57, lily hybrid (*Lilium longiflorum* × Asiatic) with the ratio of 54:46 to synthetic *Tragopogon miscellus* (*T. pratensis* × *T. dubius*) with the ratio of 49:51 (Karlov et al., 1999; Xiong et al., 2011; Chester et al., 2012). Thus, it is evident that the strength of the genome dominance depends on a cross combination and the divergence of parental genomes. Interestingly, genome dominance was also observed in another *Allium* hybrid developed from a cross between *A. fistulosum* L. and *A. cepa*. Despite previously reported problems with fertility, a number of F2 and BC1 plants (with bulb onion as pollinator) were obtained and showed the dominance toward *A. fistulosum* for three out of four isozyme markers that were tested and which showed a statistically significant violation of the theoretical 1:2:1 ratio in the F2 population (Ulloa et al., 1995). The authors hypothesized that the genome dominance might be at least partly caused by the cytoplasmic effect: their hybrids displaying *A. fistulosum* dominance were developed by pollinating *A. fistulosum* flowers with pollen from *A. cepa* and thus, the hybrids likely possessed the cytoplasm from *A. fistulosum*. However, the role of the cytoplasm could probably be ruled out in our hybrids, as the dominant genome (*A. roylei*) was used as pollinator for hybrid development. Moreover, the *A. roylei* dominance was evidenced only in F2, but not in BC1 plants.

Based on our results and other reports, it appears that genome dominance is chromosome specific. In our previous study, we found that chromosome 5 of *Festuca pratensis* (*F. pratensis*) is more prone to be replaced by its *Lolium multiflorum* (*L. multiflorum*) homoeolog in *L. multiflorum* × *F. pratensis* allotetraploids than any other chromosome (Kopecký et al., 2019). Similarly, preferential elimination of some chromosomes and a higher transmission of others were also observed in *Gossypium hirsutum* × *G. sturtianum* and *G. hirsutum* × *G. australe* hybrids (Lopez-Lavalle and Brubaker, 2007). In this study, we also found that some chromosomes or even chromosome segments of *A. cepa* are less likely to be transmitted than others (Figure 4). Specifically, chromosome 8 showed an almost equal (random) segregation, while chromosomes 2 and 3 of *A. roylei* were transmitted much more frequently to successive generation than their counterparts from *A. cepa*. Similarly, the segregation distortion was localized on all the chromosomes, except chromosomes 7 and 8 based on the results of SNP markers applied to F2 *A. cepa* × *A. roylei* (Scholten et al., 2016). This would indicate that not all the chromosomes are the subjects of the *roylei* genome dominance.

Despite the majority of the *A. cepa* genome being prone for replacement by *A. roylei*, there are two regions in *A. cepa* genome, which are transmitted at the frequency exceeding 50% in *A. cepa* × *A. roylei* hybrids. One of the regions is represented by three markers at positions 0.84, 17.49, and 19.68 cM on chromosome 5. The other segment showing > 50% transmission of *A. cepa* allele is represented by three markers at the distal part of chromosome 8 (at positions 13.65, 21.46, and 32.01 cM). One would expect a potential link between these two regions, such as *trans-*acting regulation of one by the other; however, comparison of the frequencies of the *rr*, *rc*, and *cc* classes suggests that these two regions segregate independently with no linkage. Interestingly, one would expect a segregation distortion for the segment carrying a lethal factor, previously identified at the distal end of chromosome 3 (van Heusden et al., 2000). This locus is in a close vicinity of *Pd1*, the downy mildew resistance locus. While *Pd1* is of great interest to breeders, the lethal factor, once introduced to *A. cepa* in a double dose (i.e., *rr* homozygote for this chromosome segment), is assumed to cause lethality (Scholten et al., 2007). However, we identified 8 out of 104 plants as being *rr* homozygous for all the nine markers distributed from 5.6 to 70 cM of the genetic map of chromosome 3. This might be potentially caused by double crossover of one homoeolog in the large region, where markers are absent (between 9 and 52 cM).

Recent studies have indicated that genome dominance at the chromosome level is caused by meiotic drive, a phenomenon of non-Mendelian transmission of chromosomes to the next generation. It is worth to mention differences between male and female meiosis. Male meiosis is symmetric and all the four products participate equally to successive generation. However, meiotic drivers increase the chance of the sperm cells carrying them to fertilize the eggs and, thus, violate the random transmission of sperm cells with or without the driver (Kruger and Mueller, 2021). Male meiotic drivers seem to benefit themselves and confer negative effects on the counterpart (from the other parental genome in the case of interspecific hybrids) such as reduced motility of sperms, differences in the pollinating rate of pollen grains, and failure to develop to maturity. On the contrary, female meiosis is asymmetric when one homoeolog is transmitted to the egg cell, while the other homoeolog is transferred to polar bodies, which are not participating in the next generation. This opens a way for competition between homoeologous chromosomes, where meiotic drivers act to alter the orientation of particular chromosomes in bivalents toward the developing egg cell and the polar body; in other words, chromosomes of the dominant genome are transmitted more often to egg cell and chromosomes of the submissive genome remain more frequently in polar bodies.

The first insight on the regularity of male meiosis in onion hybrids (*A. cepa* × *A. roylei*) has been provided by de Vries et al. (1992). They observed only a limited number of univalents in metaphase I and no abnormalities as bridges, fragments, or micronuclei in the later stages. Our observations fully confirm this, as bivalents were formed regularly, with both the arms paired (ring bivalents) more frequently than one (rod bivalents). The regularity of male meiosis and regular (random) transmission of chromosomes to progeny are supported by the genome composition of the BC1 generation. There were no significant differences between the numbers of parental chromosomes transmitted through pollen. Similarly, reciprocal BC1 progeny (BC1F1) of *F. pratensis* × *L. multiflorum*, where the effects of male vs. female meiosis could be studied, also showed a large difference in the genome composition. While male meiosis of hybrid produced gametes with almost equal contribution of parental chromosomes, female meiosis dramatically shifted the composition toward *L. multiflorum* (Kopecký et al., unpublished results).

Overall, our results indicate that in these hybrids, male meiosis does not contribute significantly, or not at all, to genome dominance and that it has to be female meiosis responsible for the shift toward *roylei* genome. This is in line with Courret et al. (2019) and Kruger and Mueller (2021), who hypothesized that meiotic drivers function exclusively in the male or female germline, but not both. However, we cannot completely rule out other mechanisms including differences in the gamete viability and preferential fertilization.

The mechanisms underlying the female meiotic drive have been intensively studied in hybrid mice [reviewed in Clark and Akera (2021)]. Chromosomes from the dominant genome were oriented toward the egg cell more frequently than those from the submissive genome. Molecular mechanism is so far unclear; however, the candidate is CDC42, which is signaling unequal regulation of microtubule tyrosination. This unequal tyrosination is probably caused by the difference in the copy number of kinetochore proteins between the two genomes. The abundance of the kinetochore proteins is presumably affected by the centromeric minor satellite repeats that are twice as high, while the major pericentromeric satellite repeats were almost undetectable on the chromosomes of the dominant genome (Akera et al., 2017). Hence, the dominant genome (having more copies of minor centromeric repeats and, consequently, more copies of kinetochore proteins) is preferentially transmitted to the egg cell, while the submissive genome is directed into the polar bodies. Thus, it is evident that the sequences of centromere and pericentromeric region are likely the key component of the drive (Chmatal et al., 2014). Centromeric drive has also been observed in *Drosophila* and monkey flower (*Mimulus* spp.) hybrids (Fishman and Saunders, 2008; Wei et al., 2017). Apart from the centromere, other chromosome regions in several organism were occasionally found to violate random segregation including the well-known knob-mediated meiotic drive in maize (Dawe et al., 1999). Thus, we cannot rule out other sequences outside of the native centromere and pericentromeric region to be incharge of female meiotic drive present in onion hybrids.

## Conclusion

This study reveals dominance of the *A. roylei* genome in hybrids with *A. cepa*. This dominance appears to be caused by the female meiotic drive; male meiosis seemed to be regular and produced gametes with equal proportion of chromosome from parental genomes and these chromosomes were randomly transmitted to progeny. SNP genotyping revealed that the drive had different strength across the genome, with some chromosome segments showing Mendelian segregation, while others showed statistically significant deviation from it. Meiotic drive may hamper introgression breeding in development of elite onion cultivars and cause instability of introgressed segment(s) in successive generations.

Further investigation of the centromeres/kinetochores using immunoGISH of the centromeric variant of histone H3 (CenH3) and kinetochore proteins and/or allele-specific expression profiling of genes involved in the establishment of the apparatus of the meiotic spindle may shed light on this phenomenon.

## Data Availability Statement

The original contributions presented in the study are included in the article/Supplementary Material, further inquiries can be directed to the corresponding author/s.

## Author Contributions

DK, OS, and JB conceived and designed the study. DK and JM performed GISH analyses of mitotic and meiotic chromosomes. OS made SNP genotyping. KB-M developed all the hybrids. MD provided statistical treatments. DK drafted the manuscript with contribution from OS, JM, and JB. All authors contributed to the final version of the manuscript.

## Conflict of Interest

The authors declare that the research was conducted in the absence of any commercial or financial relationships that could be construed as a potential conflict of interest.

## Publisher’s Note

All claims expressed in this article are solely those of the authors and do not necessarily represent those of their affiliated organizations, or those of the publisher, the editors and the reviewers. Any product that may be evaluated in this article, or claim that may be made by its manufacturer, is not guaranteed or endorsed by the publisher.

## Supplementary Material

Supplementary Figure 1 Meoitic configurations of *A. cepa* × *A. roylei* hybrids. GISH analysis showed regular homoeologous chromosome pairing in pachytene of prophase I **(A)** and metaphase I **(B)** and segregation of chromosomes to opposite poles during anaphase I **(C)** and reaching the poles in telophase I **(D)**. Total guide DNA (gDNA) of *A. roylei* was labeled with digoxigenin (green color) and sheared DNA of *A. cepa* was used as blocking DNA (blue color).

Supplementary Table 1 Results of genomic *in situ* hybridization (GISH) and single nucleotide polymorphism (SNP) analyses in *Allium cepa* (*A. cepa*) × *Allium roylei* (*A. roylei*) hybrids.
