# Supplementary figures and images for: Genome Dominance in Allium Hybrids (A. cepa × A. roylei)
Source: Front Plant Sci. 2022 Mar 10;13:854127. doi: 10.3389/fpls.2022.854127 (PMC8965639; doi:10.3389/fpls.2022.854127)

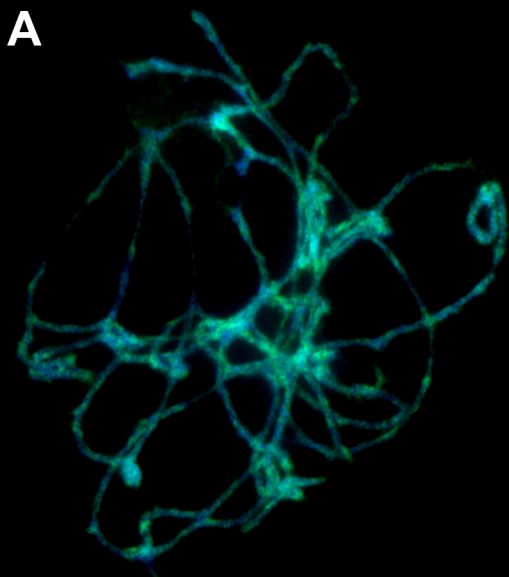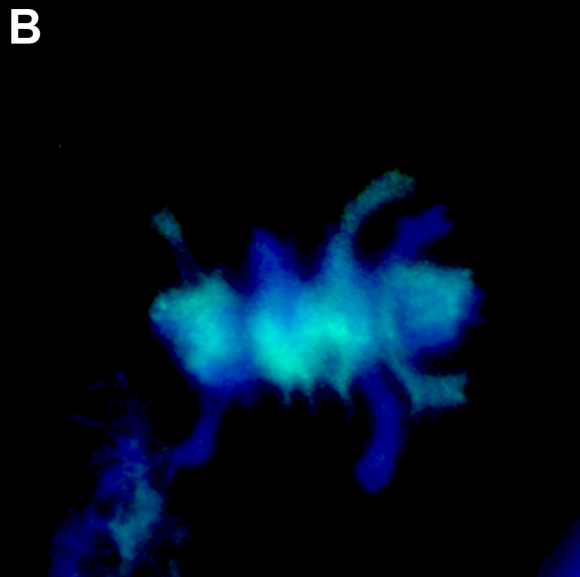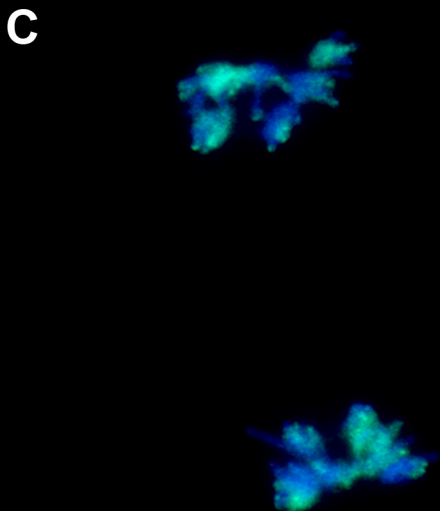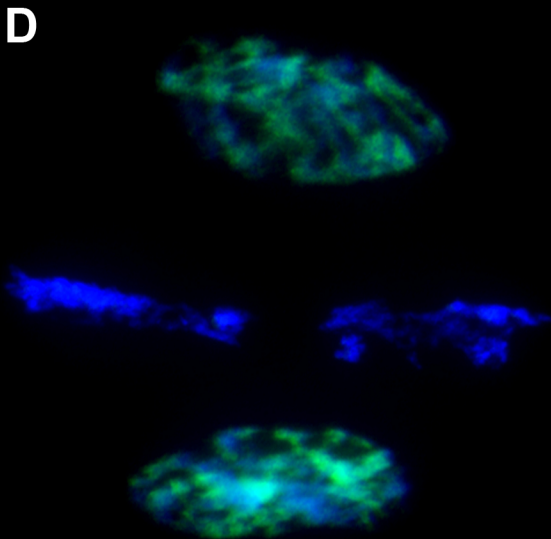

Supplement: Supplementary Figure 1 — Meoitic configurations of A. cepa × A. roylei hybrids. GISH analysis showed regular homoeologous chromosome pairing in pachytene of prophase I (A) and metaphase I (B) and segregation of chromosomes to opposite poles during anaphase I (C) and reaching the poles in telophase I (D). Total guide DNA (gDNA) of A. roylei was labeled with digoxigenin (green color) and sheared DNA of A. cepa was used as blocking DNA (blue color). [file Data_Sheet_1.PDF]
